# Evaluation of immuno-efficacy of a novel DNA vaccine encoding *Toxoplasma gondii*rhoptry protein 38 (TgROP38) against chronic toxoplasmosis in a murine model

**DOI:** 10.1186/1471-2334-14-525

**Published:** 2014-09-30

**Authors:** Ying Xu, Nian-Zhang Zhang, Qi-Dong Tan, Jia Chen, Jing Lu, Qian-Ming Xu, Xing-Quan Zhu

**Affiliations:** State Key Laboratory of Veterinary Etiological Biology, Key Laboratory of Veterinary Parasitology of Gansu Province, Lanzhou Veterinary Research Institute, Chinese Academy of Agricultural Sciences, Lanzhou, Gansu Province, 730046 P.R. China; College of Animal Science and Technology, Anhui Agricultural University, Hefei, Anhui Province, 230036 P.R. China

**Keywords:** *Toxoplasma gondii*, Toxoplasmosis, TgROP38, DNA vaccine, Protective immunity, Mouse

## Abstract

**Background:**

*Toxoplasma gondii* is an obligate intracellular parasite which can infect almost all mammalian animals, leading to toxoplasmosis. *T. gondii* rhoptry protein 38 (TgROP38) is an active rhoptry protein kinase which is involved in the inhibitory effect on host cell transcription by down-regulating the MAPK signaling track.

**Methods:**

TgROP38 gene was amplified and inserted into eukaryotic vector pVAX I and formed the DNA vaccine pVAX-ROP38. Mice in the experimental group were intramuscularly immunized with pVAX-ROP38 and those injected with pVAX I, PBS or nothing were treated as controls. After three injections at two week intervals, all mouse groups were challenged intraperitoneally with 1000 tachyzoites of the virulent *T. gondii* RH strain (Type I, ToxoDB #10) and 10 cysts of the PRU strain (Type II, ToxoDB #1), respectively.

**Results:**

Mice inoculated with pVAX-ROP38 vaccine had a higher level of IgG antibodies (*P* < 0.01) and T lymphoproliferative response. The high ratio of IgG2a/IgG1 and the increasing levels of IFN-γ and IL-2 (*P* < 0.05) indicated an activated Th1 cell-mediated immune responses. Furthermore, the CD4^+^ and CD8^+^ proportions in vaccinated mice were also increased significantly compared with that in mice of the three control groups (*P* < 0.01). In the model of acute infection, the average survival time of mice in the pVAX-ROP38 group (8.1 days ± 0.75) was no statistically different compared to that in the PBS, pVAX I and blank control groups which died within 7 days. However, in the model of chronic infection, the brain cyst reduction in the pVAX-ROP38 group reached 76.6%, compared to controls (*P* < 0.01).

**Conclusions:**

The present study revealed that the pVAX-ROP38 vaccine could elicit strong humoral and cell immunity response against chronic *T. gondii* infection in mice, resulting in the reduction of the brain cyst formation effectively, which suggests that TgROP38 is a desirable vaccine candidate against chronic *T. gondii* infection.

**Electronic supplementary material:**

The online version of this article (doi:10.1186/1471-2334-14-525) contains supplementary material, which is available to authorized users.

## Background

The obligate intracellular protozoa parasite *Toxoplasma gondii* can infect virtually all warm-blooded hosts including wild mammals, birds, livestock, poultry and human beings throughout the world, posing a significant public health concern [1, 2]. The symptoms of *T. gondii* infection in humans ranged from asymptomatic in immunocompetent individuals to devastating in immune-compromised individuals and unprotected fetuses [2–4]. Epidemiologic survey results showed that the high *T. gondii* prevalence in many economic animals led to considerable economic losses [5–7].

Several chemical drugs such as sulphadiazine, atovaquone and pyrimethamine could only control *T. gondii* acute infection with few adverse effects, but can not eliminate chronic infection [2, 8, 9]. Using vaccines, as the successful application in prevention of hepatitis B (HBV) or Bacille Calmette-Guerin (BCG), is thus of high priority [10], but so far only one attenuated-live *T. gondii* vaccine was available for veterinary uses, and no vaccine is available for defending against *T. gondii* infection in humans [11, 12]. DNA vaccines were considered to be more efficient and safer than live and attenuated-live vaccines, as they were able to elicit long-lasting humoral and cell-mediated immune responses, and significant progress has been made in the identification of candidate antigens against *T. gondii* in the last several years [12, 13].

Rhoptry proteins (ROPs) were shown to exist on the parasitophorous vacuole membrane and were also identified to be the virulence factors participating in *T. gondii* invasion [14, 15]. Due to their key biological roles, *T. gondii* rhoptry proteins 5, 13, 16 and 18 (TgROP5, TgROP13, TgROP16 and TgROP18) have been proven as potential vaccine candidates [16–19]. *T. gondii* rhoptry protein 38 (TgROP38) is predicted to be an active rhoptry protein kinase (ROPK) that could have an inhibitory effect on host cell transcription by down-regulating the MAPK signaling track [20–22]. TgROP38 is observed inside rhoptries and is dramatically expressed among *T. gondii* genotypes (up 64 times in VEG strain, up 8 times in ME49 strain compared with the RH strain) [20, 21]. Furthermore, the low sequence variation in TgROP38 gene among different *T. gondii* strains implied that TgROP38 may represent a potential vaccine candidate against *T. gondii* infection [23].

The objectives of the present study were to examine the immunogenicity of the recombinant eukaryotic expression plasmid pVAX-ROP38 and to evaluate the acquired immuno-efficacy of this DNA vaccine against challenge infection with highly virulent RH strain (Type I, ToxoDB #10) and less virulent PRU strain (Type II, ToxoDB #1) of *T. gondii* in a mouse model.

## Methods

### Mice and parasites

Specific-pathogen-free (SPF) grade female Kunming mice aged six to eight weeks old were purchased from Lanzhou University Laboratory Animal Center (Lanzhou, Gansu Province, China). All mice were fed with basal diet and tap water *ad libitum* in strict accordance with Good Animal Practice requirements of the Animal Ethics Procedures and Guidelines of the People’s Republic of China, and the study was approved by the Animal Ethics Committee of Lanzhou Veterinary Research Institute, Chinese Academy of Agricultural Sciences (Approval No. LVRIAEC2012-011).

The PRU strain was kept in Kunming mice by oral passage of 10 brain cysts. Tachyzoites of the RH strain were preserved in liquid nitrogen in the Department of Parasitology, Lanzhou Veterinary Research Institute, Chinese Academy of Agricultural Sciences (Lanzhou, Gansu Province, China), and harvested from the peritoneal fluids through serial intraperitoneal passage in Kunming mice. The tachyzoites were also used for preparing soluble tachyzoite antigens (STAg) by ultrasonication after washing by sterile phosphate-buffered saline (PBS). The lysate was then centrifuged at 10, 000 × *g* for 15 min and the supernatant was kept at -80°C until further use.

### Construction of the eukaryotic expression plasmids

The complete ROP38 gene sequence of ME49 strain in ToxoDB database (TGME49_242110) was used to design specific primers ROP38U1 (5′-CGG*GGTACC*ATGAAAAATACTCTGTTGTCA-3′) and ROP38D1 (5′- TGC*TCTAGA*TCAAAATTGATGCGTTCTTAT-3′), in which *Kpn* I and *Xba* I recognition sites were introduced in both primers and underlined. The ORF of TgROP38 gene was amplified by PCR using genomic DNA (gDNA) from *T. gondii* RH strain as template. The PCR product was purified by using TIANquick Midi Purification Kit (TIANGEN, Beijing, China). The purified PCR product and vector pVAX I (Invitrogen, Carlsbad, California, USA) were then cleaved by *Kpn* I and *Xba* I. The TgROP38 fragment was then inserted into vector pVAX I using T4 ligase enzyme, and formed pVAX-ROP38. The concentration of the recombinant plasmids was determined using a spectrophotometer at OD_260_ and OD_280_. The plasmids were diluted with PBS to a final concentration of 1 μg/μl and stored at -20°C.

### Expression of pVAX-ROP38 *in vitro*

The recombinant plasmid pVAX-ROP38 and empty vector pVAX I were transfected into HEK 293 cells, respectively, using lipofectamine 2000 reagent (Invitrogen) according to the manufacturer’s instructions. Indirect immunofluorescence assay (IFA) was used to detect the expression of pVAX-ROP38 at 48 hours as described previously [24].

### Construction of the prokaryotic expression plasmids

To amplify TgROP38 gene and ligate to the prokaryotic expression vector pET-30a(+), *Kpn* I and *Bam*H I recognition sites were introduced in the primers. Then both PCR products and the pET-30a(+) were digested by *Kpn* I and *Bam*H I, respectively. The extracted TgROP38 gene fragment was linked into purified linear vector pET-30a(+) by T4 DNA ligase (TaKaRa, Dalian, Liaoning Province, China), formed the pET-ROP38 recombinant plasmid.

### Expression of TgROP38 protein in *E. coli*BL21 (DE3)

The obtained recombination plasmids were transferred into *E. coli* BL21 (DE3) competence cell and grown in Luria Bertani (LB) with kanamycin (25 μg/ml). The recombinant TgROP38 (rTgROP38) protein was expressed under the condition of 1.0 mM IPTG (Sangon, Shanghai, China) and shaking for 8 hours at 37°C. Then the protein was purified using Ni-NTA His bind resin (Novagen, Shanghai, China) according to the manufacturer’s instructions. The purified protein was examined by 10% sodium dodecyl sulfare polyacrylamide gel electrophoresis (SDS-PAGE), and then diluted by sterile PBS to a final concentration of 10 μg/ml and stored at -80°C.

### Immunization and challenge

Three groups of mice (28 per group) were injected intramuscularly with 100 μl diluted pVAX-ROP38 DNA vaccine, PBS or empty pVAX I plasmid, respectively. Another group of 28 mice were kept as blank control without injection. The last three groups were served as control groups. Mice in the pVAX-ROP38 DNA vaccine, PBS and pVAX I groups were vaccinated three times with a two-week interval. The blood of mice in each group was collected from mouse tail vein before each immunization and the sera were separated and stored at -20°C for detection of specific antibodies.

Two weeks after the last immunization, 10 mice in each group were challenged intraperitoneally with 1000 tachyzoites of the virulent *T. gondii* RH strain, and other 6 mice were inoculated with 10 cysts of the attenuated virulent PRU strain orally. Mice injected with RH strain were observed twice a day for mortality until a fatal outcome for all animals. The brain cysts were determined one month after the challenge infection. Each brain was homogenized in 2 ml PBS, and the mean number of cysts per brain was calculated.

### Immunoblotting analysis of the recombinant protein

The purified rTgROP38 protein was identified by western blotting after 10% SDS-PAGE. The protein was semi-dry transferred onto nitrocellulose (NC) membranes (Pall, New York, USA) at 15 V for 10 min. Then nonspecific binding sites were blocked with 5% bovine serum albumin (BSA) in PBST (PBS plus 0.05% Tween-20) for 1 h at room temperature (RT). The NC membranes were then incubated for 1 h at RT with the sera of immunized mice on week 6 (diluted in 1:200) and non- immunized sera on week 0 as negative control, respectively. After being washed 3 times with PBST, the membranes were incubated with diluted secondary antibody (goat anti-mouse IgG-HRP, Sigma, St. Louis, Missouri, USA; 1: 5000) for 1 h at RT. Proteins were visualized with 4-chloro-1-naphthol (4-CN; Sangon) according to the manufacturer’ instruction.

### Evaluation of humoral response to pVAX-ROP38

Levels of antigen-specific IgG, IgG1 and IgG2a antibodies in mouse serum samples were detected by ELISA using SBA Clonotyping System-HRP Kit (Southern Biotech Co., LTD, Birmingham, Alabama, USA). A total of 100 μl rTgROP38 (10 μg/ml) was coated on 96-well microtiter plates at 4°C overnight. Mouse serum samples were added to the wells and incubated at RT for 1 h with gentle shaking. Then each well was incubated with 100 μl of horseradish-peroxidase (HRP) conjugated anti-mouse IgG diluted at 1:250, anti-mouse IgG1 or IgG2a (1: 500) as secondary antibodies for 1 h with gentle shaking. After adding 3, 3′, 5, 5′-Tetramethylbenzidine substrate solution (TMB) (5% TMB; 0.03% H_2_O_2_) for 15 min, the reaction was terminated by adding 2 M H_2_SO_4_ stopping solution and the absorbance was measured at 450 nm. All measurements were performed in triplicate.

### Splenocytes proliferation analysis by MTS

Two weeks after the last immunization, three mice of each group were euthanized and their spleens were removed aseptically. A single-cell suspension of splenocytes was prepared as described previously [17]. Cells were then covered in 96-well microtiter plates (Costar, Washington DC, USA) at a density of 5 × 10^5^ cells per well, and cultured with Concanavalin A (ConA; 5 μg/ml; Sigma) or STAg (10 μg/ml) or medium alone, respectively. After incubation at 37°C in a 5% CO2 incubator, the proliferative activity was measured by MTS method (Promega, Madison, Wisconsin, USA) four days later. All assays were performed in triplicate. The stimulation index (SI) was calculated by using the formula (OD_570STAg_/OD_570M_): (OD_570ConA_/OD_570M_).

### Identification of CD4^+^ and CD8^+^T cells by flow cytometry analysis

The percentage of T cell subclasses including CD4^+^ and CD8^+^ in spleen were determined by flow cytomety analysis. With staining by phycoerythrin (PE)-labeled anti-mouse CD3 (eBioscience, San Diego, California, USA), allophycocyanin (APC)-labeled anti-mouse CD4 (eBioscience) and fluorescein isothiocyanate (FITC)-labeled anti-mouse CD8 (eBioscience) antibodies, the single-cell suspension splenocytes were washed by PBS, the cells were then fixed with FACScan buffer (PBS containing 1% FCS and 0.1% Sodium azide) and 2% paraformaldehyde. All samples were analyzed using SYSTEM II software through FACScan flow cytometer (BD Biosciences, San Jose, California, USA).

### Cytokine assays

The splenocyte suspension from each group was co-cultured with STAg in a 5% CO2 incubator at 37°C, and the supernatants were collected at 24 h, 24 h, 72 h and 96 h for determining the level of IL-2, IL-4, IL-10 and IFN-γ, respectively. All of the samples were examined in triplicate using commercial ELISA kits following the manufacturer’s instructions (BioLegend, San Diego, California, USA).

### Statistical analysis

Data are presented as the mean ± SD. All statistical analyses including antibody responses, splenocytes proliferation assays, flow cytometric assays, cytokine assays and survival time were carried out by the two-way AVONA test, GraphPad Prism5.0® (GraphPad Software, Inc., San Diego, California, USA). *P* value of less than 0.05 or 0.01 was considered significantly different or highly significant different, respectively.

## Results

### Eukaryotic and prokaryotic expression of TgROP38 protein

**Figure 1:**
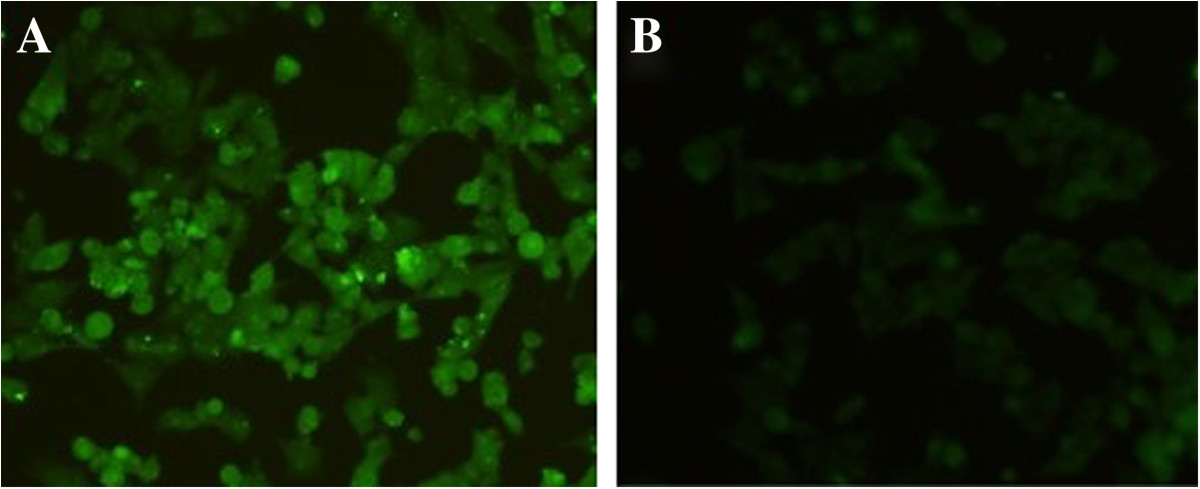
**The indirect immunofluorescence assay (IFA) analysis of** ***Toxoplasma gondii*** **rhoptry protein 38 (TgROP38). (A)** HEK 293 cells were transfected with pVAX-ROP38; **(B)** empty vector pVAX I.

Specific green fluorescence in the HEK 293 cells transfected with the recombinant plasmid was observed, but not in the negative controls transfected with the same amount of empty pVAX I (Figure 1), showing that recombinant TgROP38 protein was expressed in HEK 293 successfully.

**Figure 2:**
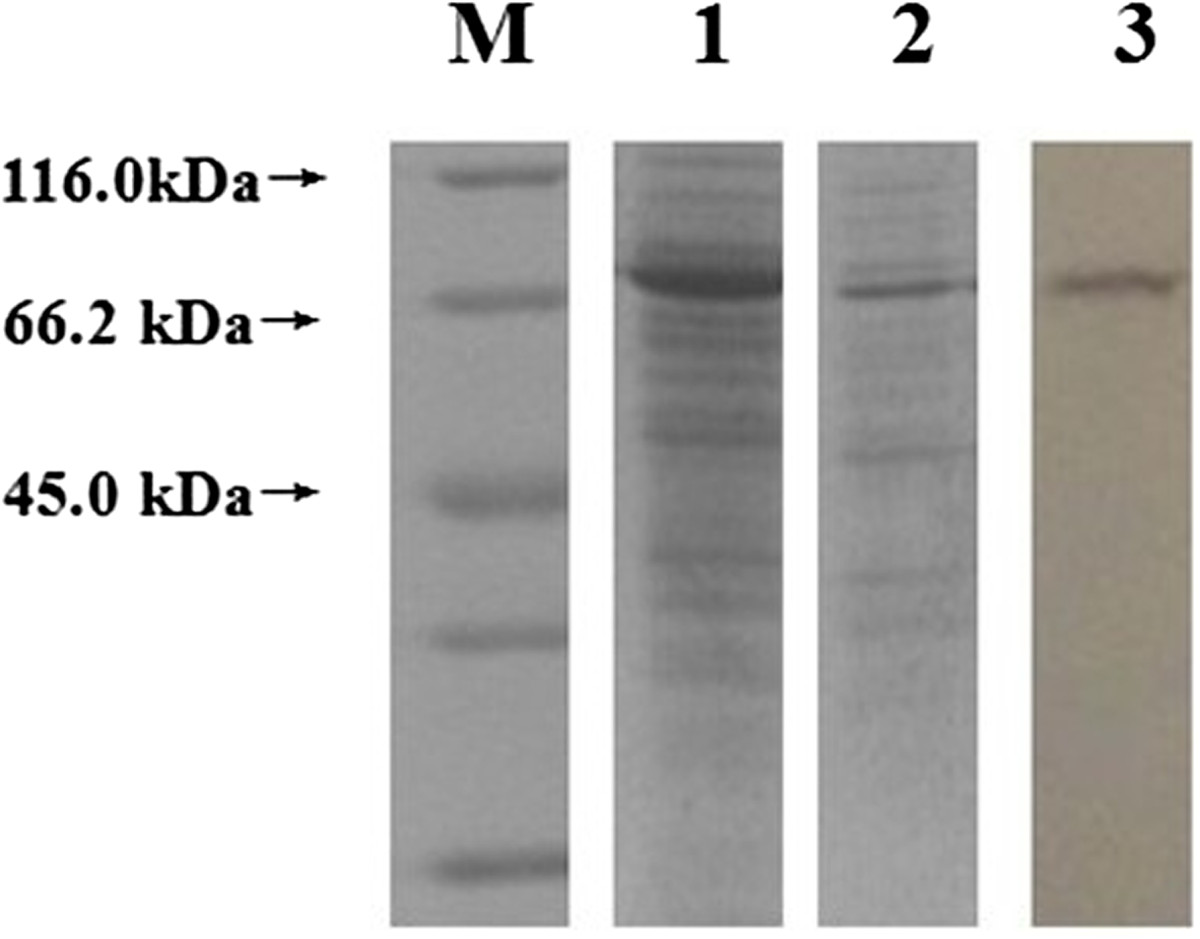
**Identification of recombinant rhoptry protein 38 (rTgROP38) of** ***Toxoplasma gondii*** **by SDS-PAGE and Western-blot.** M: unstained protein marker; 1: Fusion protein TgROP38 expressed by pET-ROP38 recombinant plasmid; 2: Purified fusion protein TgROP38; 3: Western-blot analysis of fusion protein TgROP38.

The recombinant plasmid pET-ROP38 was expressed in *E. coli* BL21 (DE3) and examined by SDS-PAGE and western-blot. The results showed that the rTgROP38 protein was approximately 66 kDa, which was consistent to the expected molecular weight, and was recognized by anti-ROP38 antibody (Figure 2).

### Antibody detection

**Figure 3:**
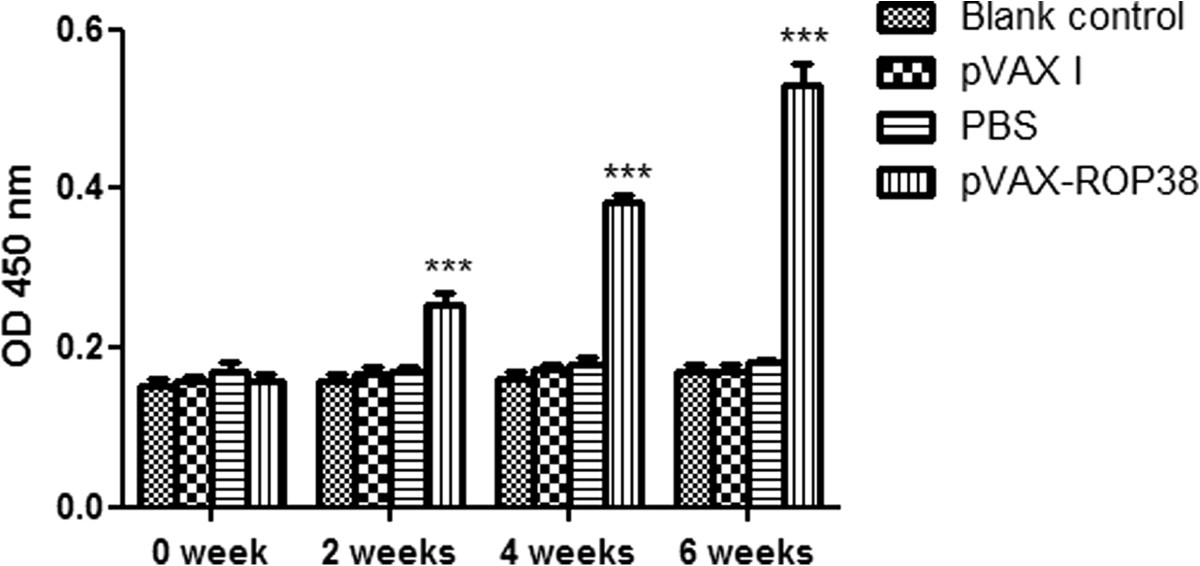
**Detection of specific IgG antibodies in mice after immunization with** ***Toxoplasma gondii*** **rhoptry protein 38 (TgROP38).** Serum samples were detected at 2 weeks interval by ELISA. Data are presented as the mean ± SD (n = 3). *** represents highly significantly differences (*P* < 0.01) compared with the control groups.

The levels of specific antibodies IgG, IgG1 and IgG2a were detected by ELISA. Compared with the three control groups, a statistically significantly higher level of IgG antibody was detected in the sera of mice immunized with pVAX-ROP38 (*P* < 0.01), and the OD values of IgG were continuously increased with successive DNA immunization. There were no statistically significantly differences in IgG levels among the three control groups (Figure 3).

**Figure 4:**
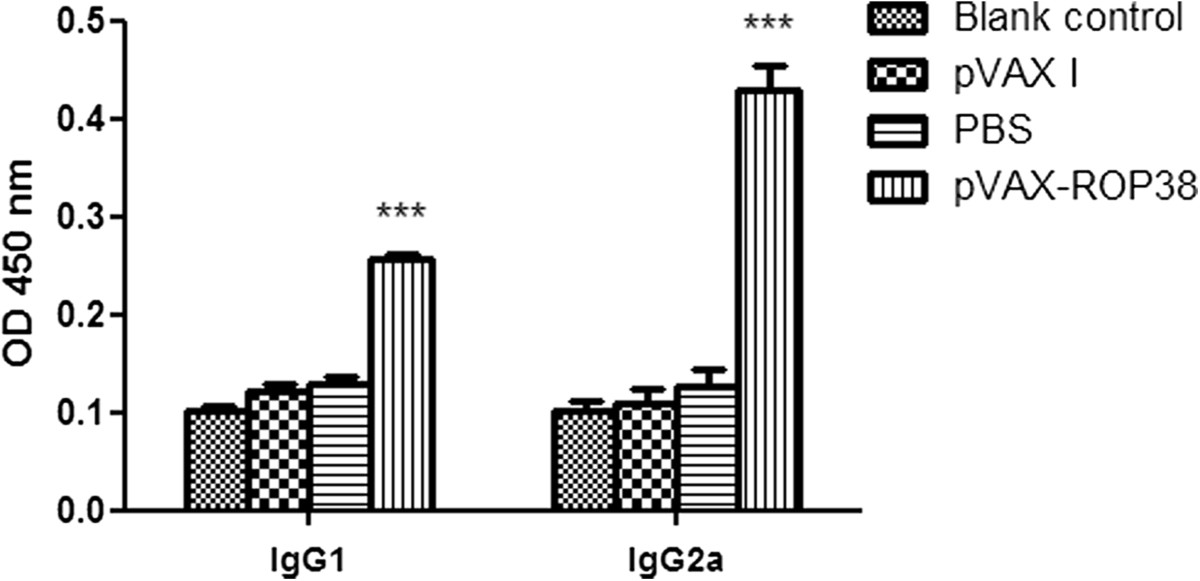
**Specific IgG1 and IgG2a pattern upon immunization with pVAX-ROP38.** Data are presented as the mean ± SD (n = 3). *** represents highly significantly differences (*P* < 0.01) compared with the control groups.

The levels of IgG1 and IgG2a in the experimental group (immunized with TgROP38) were also the highest in comparison with that of the three control groups (*P* < 0.01) (Figure 4). The type of immune response induced by the pVAX-ROP38 was evaluated by the ratio of IgG1/IgG2a. The results showed that a significantly higher IgG2a value than IgG1 from the sera collected on week 6 was detected in mice immunized with pVAX-ROP38, while no significantly differences were observed in the controls (Figure 4), demonstrating that a Th1-type cell immune response was elicited by immunization with pVAX-ROP38.

### Splenocyte proliferation

Proliferation SI measured at OD_570nm_ in mice vaccinated with pVAX-ROP38 (0.90 ± 0.02) was similar to that immunized with PBS (0.91 ± 0.01), pVAX I (0.89 ± 0.07) and blank control (0.97 ± 0.01) (*P* > 0.05) (Table 1).

**Table 1:** **Proliferation SI of mice in each group**

Groups	SI
Blank control	0.97 ± 0.01
pVAX I	0.89 ± 0.07
PBS	0.91 ± 0.01
pVAX-ROP38	0.90 ± 0.02

Data are presented as the mean ± SD (n = 3). The stimulation index (SI) was calculated by using the formula (OD_570STAg_/OD_570M_): (OD_570ConA_/OD_570M_).

### Evaluation of the percentages of CD4^+^ and CD8^+^of T lymphocytes

After third immunization, the percentage of CD3^+^ CD4^+^ CD8^-^ and CD3^+^ CD8^+^ CD4^-^ T lymphocytes in the pVAX-ROP38 group were significantly higher than those in the control groups, respectively (*P* < 0.01) (Table 2). But there were no significantly differences in the ratio of CD8^+^/CD4^+^ between mice immunized with pVAX-ROP38 and in controls (*P* > 0.05).

**Table 2:** **CD4** ^**+**^ **and CD8** ^**+**^ **of T lymphocytic subclasses**

Groups	CD3^+^CD4^+^CD8^-^	CD3^+^CD8^+^CD4^-^	Ratio
Blank control	15.3 ± 1.9	5.0 ± 0.4	3.0 ± 0.2
pVAX I	14.2 ± 1.9	6.5 ± 1.2	2.2 ± 0.5
PBS	13.7 ± 2.4	6.4 ± 1.3	2.2 ± 0.2
pVAX-ROP38	29.3 ± 1.1^***^	13.6 ± 0.8^***^	2.2 ± 0.1

Samples were detected by flow cytometric assays, and data are presented as the mean ± SD (n = 3).

***represents statistically highly significant difference (*P* < 0.01).

### Cytokine production

The cytokines produced by spleen cells from the four groups of mice were evaluated by ELISA. The results showed a significantly high level of both IFN-γ and IL-2 in the pVAX-ROP38 group compared with that in the three control groups (*P* < 0.05). However, the level of IL-10 from spleen cells of the mice immunized with pVAX-ROP38 was significantly decreased compared to those in control groups (*P* < 0.01) (Table 3).

**Table 3:** **Cytokine production of splenocytes induced by STAg**

Groups	Cytokine production			
	IFN-γ	IL-2	IL-4	IL-10
**Blank control**	47.3 ± 16.6	12.7 ± 8.0	11.6 ± 9.5	477.3 ± 87.5
**pVAX I**	34.9 ± 11.2	12.4 ± 11.8	12.1 ± 3.4	322.5 ± 179.2
**PBS**	36.4 ± 4.8	11.3 ± 6.0	13.3 ± 1.8	356.1 ± 175.3
**pVAX-ROP38**	575.2 ± 123.0^***^	195.3 ± 28.4^*^	< 5	48.7 ± 24.3^***^

*STAg* soluble tachyzoite antigens.

Data are presented as the mean ± SD (n = 3).

*represents statistically significant difference (*P* < 0.05).

***represents statistically highly significant difference (*P* < 0.01).

### Protection against *T. gondii*challenge

**Figure 5:**
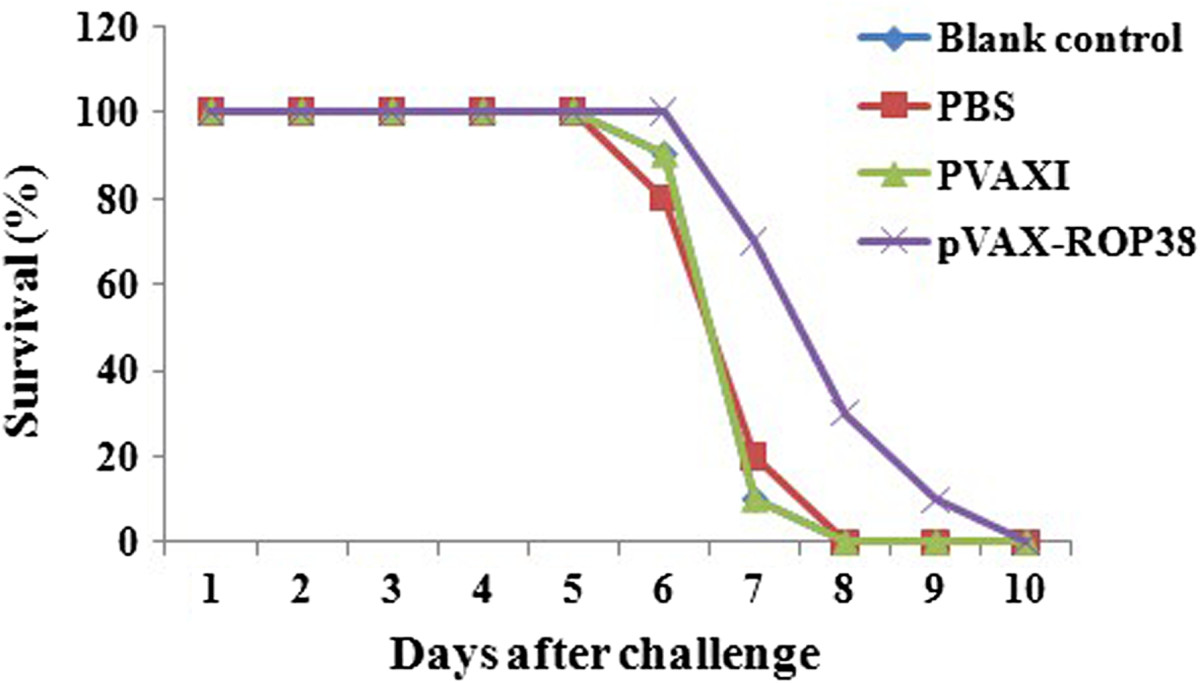
**Survival curve of mice after challenge infection with** ***Toxoplasma gondii*** **RH strain.** Mice were challenged with 1000 tachyozoites of the RH strain intraperitoneally two weeks after the third immunization.

The survival rate of mice after acute *T. gondii* infection in the four groups is shown in Figure 5. After challenge infection intraperitoneally with a fatal dose of tachyzoites of the virulent *T. gondii* RH strain, the average survival time of mice in the pVAX-ROP38 group was slightly longer than that of the three control groups, but the difference was not statistically significant (*P* > 0.05) (Figure 5). The mice received PBS and pVAX I showed similar survival time compared to the blank control group (Figure 5).

The number of brain cysts in mice of the four groups is showed in Table 4. The number of brain cysts in mice from the experimental group (immunized with pVAX-ROP38) was significantly decreased (76.6%) compared to that of the three control groups (*P* <0.01).

**Table 4:** **Mean cyst burden per mouse brain 4 weeks after challenge with 10 cysts of** ***Toxoplasma gondii*** **PRU strain per mouse**

Groups	Number of brain cysts
Blank control	3231.7 ± 533.6
pVAX I	3488.9 ± 914.7
PBS	3166.7 ± 671.7
pVAX-ROP38	755.6 ± 1870.0^***^

Data are presented as the mean ± SD (n = 6).

***represents statistically highly significant difference (*P* < 0.01).

## Discussion

*T. gondii* is an important zoonotic Apicomplexan parasite, but no drugs could eliminate the pathogen from the host effectively. In recent studies, DNA vaccines have shown the potential to defend against *T. gondii* infection in view of their abilities to induce long-term humoral and cellular immune responses in animal models. Many rhoptry proteins (ROP5, ROP13, ROP16 and ROP18) [16–19] are identified to be potential candidates for development of *T. gondii* DNA vaccines. TgROP38, a new member of the rhoptry protein family, was firstly identified by the phylogenomic approach and was found to regulate the expression of host transcription factors, signaling pathways and cell proliferation, and apoptosis that sum up about 1200 host genes [21]. These key biological roles of TgROP38 in *T. gondii* infection of the host have stimulated us to evaluate whether TgROP38 could elicit effective immune responses against infection with *T. gondii* in the mice model. Therefore, we constructed the recombinant plasmid pVAX-ROP38 and evaluated its vaccinal potentiality and the acquired immune responses it elicited.

Immunization of mice with pVAX-ROP38 induced significantly reduction of brain cysts (76.6%) in the vaccinated mice compared to the controls, and this efficacy is better than other single gene vaccines including TgIF2α (44.1%) [25] and TgCDPK3 (50%) [24]. However, immunization of mice with pVAX-ROP38 had virtually no effects on the prolonged survival time of mice in the pVAX-ROP38 group following challenge infection with the highly virulent RH strain. This is likely due to the low expression of TgROP38 in tachyzoites, thus the relatively low responsivity to tachyzoited TgROP38 induced by immunization with pVAX-ROP38 failed to prevent *T. gondii* RH strain invasion in the mice model. However, the expression of TgROP38 gene is up-regulated in the bradyzoite stage [20, 21], and it is speculated that TgROP38 participates in the tissue cyst formation, thus contributes to the significant decrease of brain cysts in pVAX-ROP38 vaccinated mice after challenge with PRU strain.

The specific antibodies against *T. gondii* can inhibit the parasite to attach to the host cell receptors, and can promote macrophages to kill intracellular parasites, which seem to be important in controlling *T. gondii* infection and preventing reactivation [26]. In the present study, the high level of specific IgG antibody against TgROP38 was induced in the experimental group (pVAX-ROP38 vaccinated mice) compared with three control groups (*P* < 0.01). Further analyses of the ratio of IgG1/ IgG2a revealed a higher level of IgG2a, indicating that pVAX-ROP38 could elicit the Th1-biased immune response, which is considered to play a critical role in the protective immunity against *T. gondii*[27, 28].

Among the subclasses of T lymphocytes, CD4^+^ and CD8^+^ T lymphocytes play an important role in host resistant to *T. gondii* infection. The CD3^+^ CD4^+^ CD8^-^ is the surface marker of T helper (T_H_) cells that can participate in the adaptive immune responses, while CD3^+^ CD8^+^ CD4^-^ is expressed on cytotoxic T cells (CTLs) which is classified as a pre-defined cytotoxic role within the immune system [29]. Also, it has been demonstrated that CD8^+^ T cells are pivotal for long-term protection in murine models of chronic toxoplasmosis [30].

In this study, both CD3^+^ CD4^+^ CD8^-^ and CD3^+^ CD8^+^ CD4^-^were significantly increased in the vaccinated mice (*P* < 0.01) compared to the controls with a slightly higher ratio of CD3^+^ CD8^+^ CD4^-^/CD3^+^ CD4^+^ CD8^-^, indicating that DNA immunization with pVAX-ROP38 could elicit a synergistic effect on CD4+ helper T-lymphocytes and CD8+ and cytotoxic T-lymphocytes. CD8^+^ T cells can control *T. gondii* infection through the production of inflammatory cytokines. The increased levels of IFN-γ in the mice immunized with pVAX-ROP38 contribute to induce protective immunity against infection with *T. gondii* PRU strain.

IFN-γ could differentiate naive CD4^+^ T cells (Th0 cells) into Th1 cells, while suppress Th2 cell differentiation [31, 32]. The cytokine is secreted by T_H_ cells, CTL cells or NK cells and has antiviral, immunoregulatory and anti-tumor functions [33]. When antigens bind to the T cell receptors (TCRs), IL-2 is stimulated to secrete especially in adaptive immunity, thus IL-2 regulates the growth, differentiation and survival of antigen-specific CD4^+^ T cells and CD8^+^ T cells and participates in the development of immunological memory [34, 35]. The present study observed significantly increased levels of IFN-γ and IL-2 in pVAX-ROP38 immunized mice. IL-4 can up-regulate MHC class II production [36], and IL-10 inhibits B and T lymphocytes proliferation and suppresses human macrophages activity *in vivo*[37–39]*.* The sharply increased levels of IFN-γ and IL-2 and the decreased levels of IL-4 and IL-10 cytokines in mice immunized with pVAX-ROP38 demonstrated that a strongly Th1-type immune response was elicited.

## Conclusion

In summary, this study revealed that DNA vaccine pVAX-ROP38 encoding TgROP38 can trigger strong humoral and cellular immune responses, and can protect against the formation of *T. gondii* brain cysts. TgROP38 warrants further studies as a potential vaccine candidate against chronic *T. gondii* infection*.*

## Authors’ original submitted files for images

Below are the links to the authors’ original submitted files for images. Authors’ original file for figure 1 Authors’ original file for figure 2 Authors’ original file for figure 3 Authors’ original file for figure 4 Authors’ original file for figure 5
