# Supplementary figures and images for: Evaluation of immuno-efficacy of a novel DNA vaccine encoding Toxoplasma gondiirhoptry protein 38 (TgROP38) against chronic toxoplasmosis in a murine model
Source: BMC Infect Dis. 2014 Sep 30;14:525. doi: 10.1186/1471-2334-14-525 (PMC4261603; doi:10.1186/1471-2334-14-525)

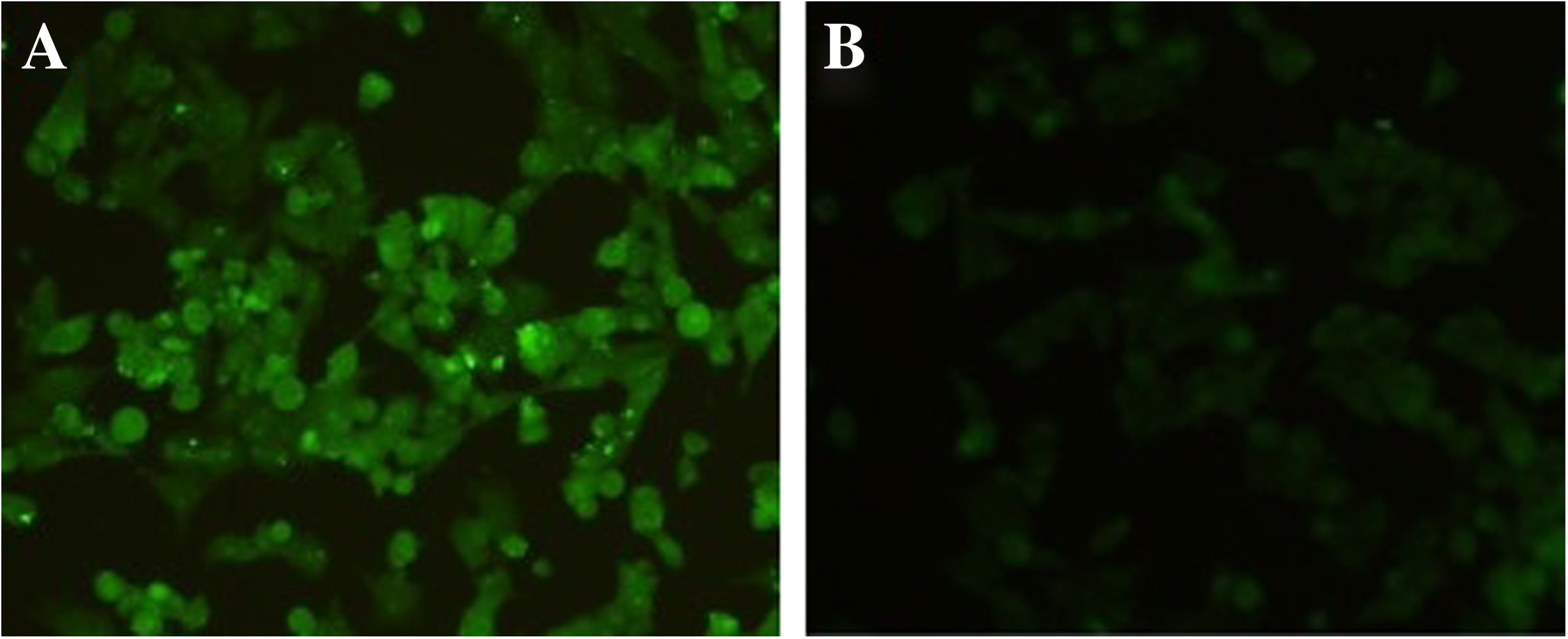

Supplement: Supplementary file 1 — Authors’ original file for figure 1 [file 12879_2014_3845_MOESM1_ESM.tif]

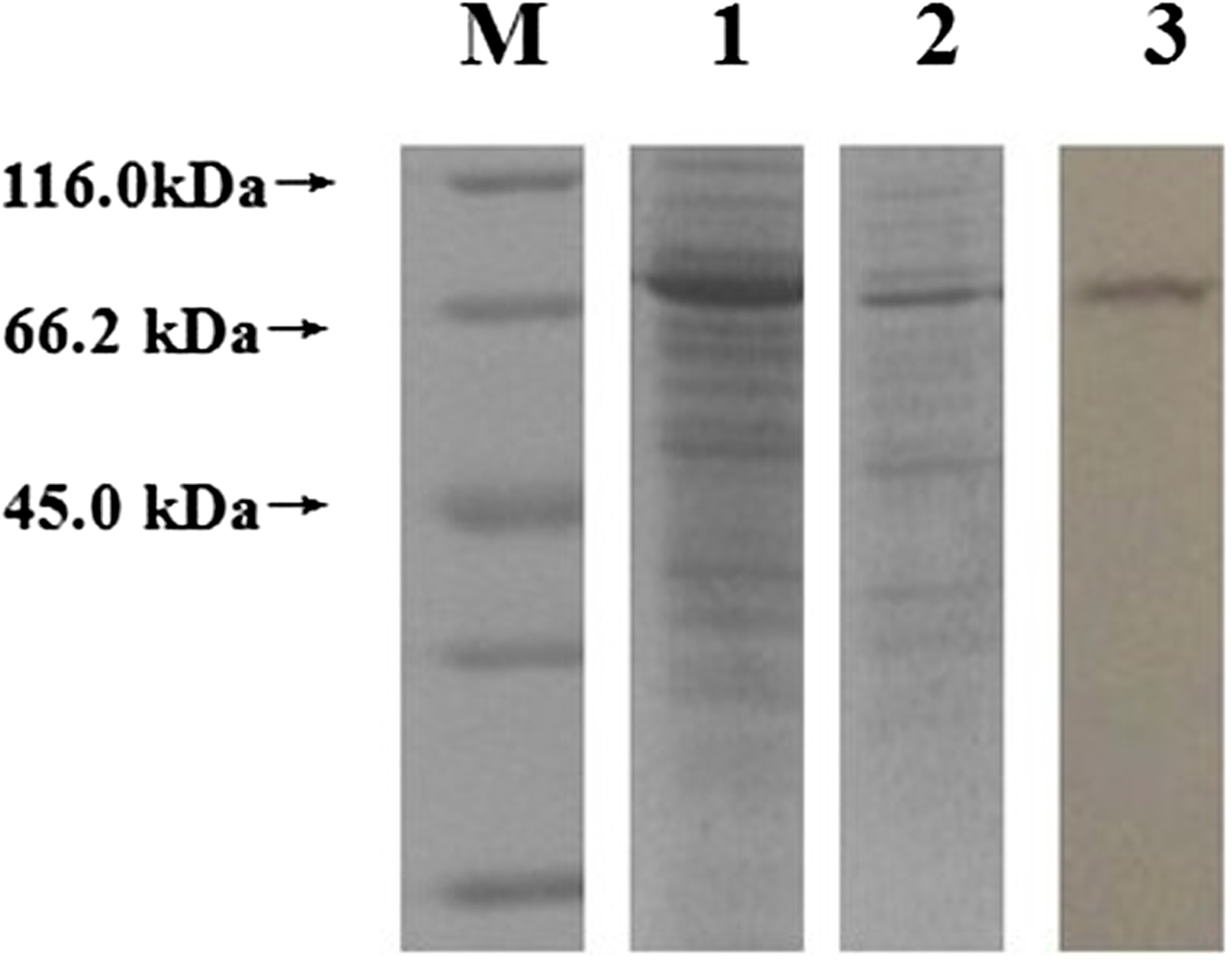

Supplement: Supplementary file 2 — Authors’ original file for figure 2 [file 12879_2014_3845_MOESM2_ESM.tif]

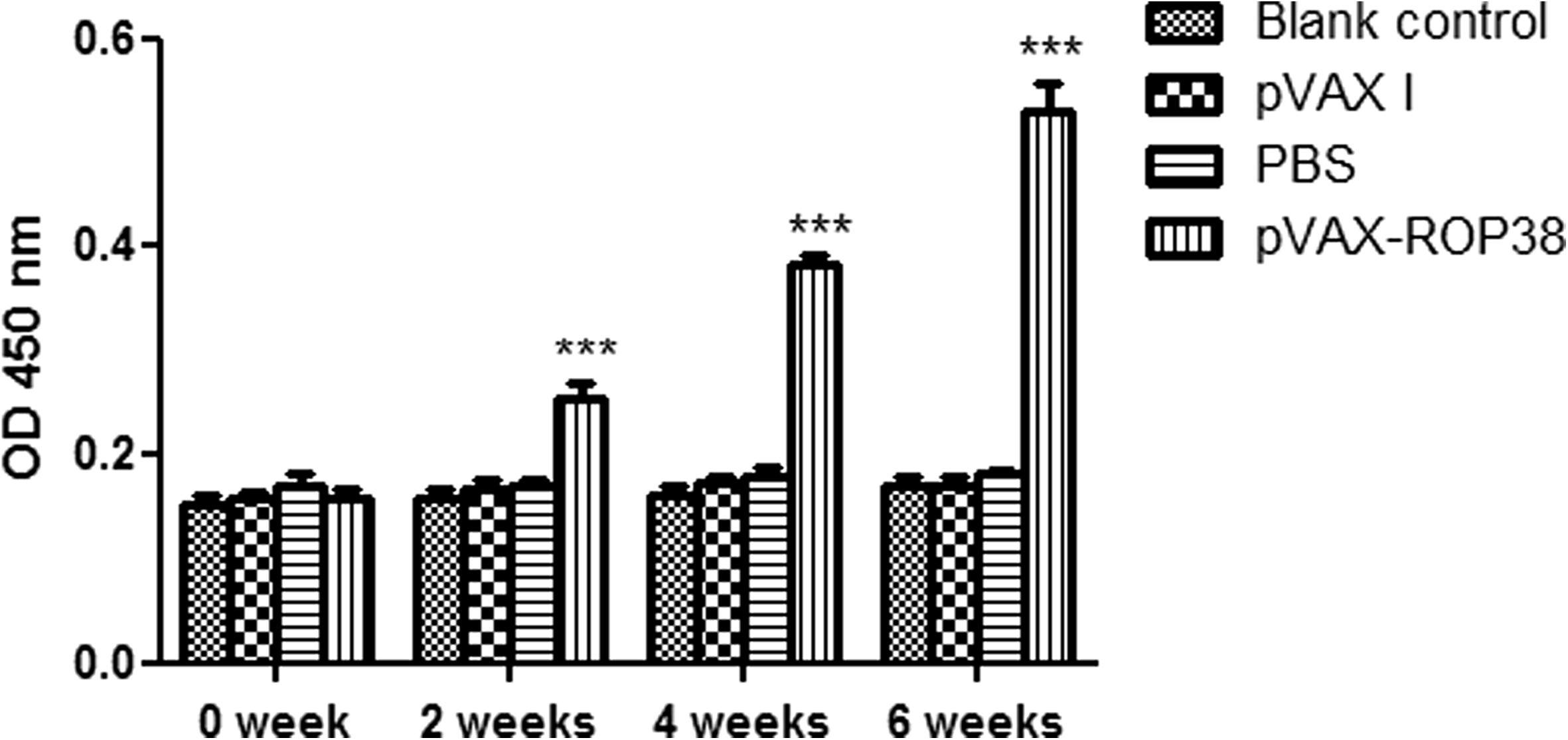

Supplement: Supplementary file 3 — Authors’ original file for figure 3 [file 12879_2014_3845_MOESM3_ESM.tif]

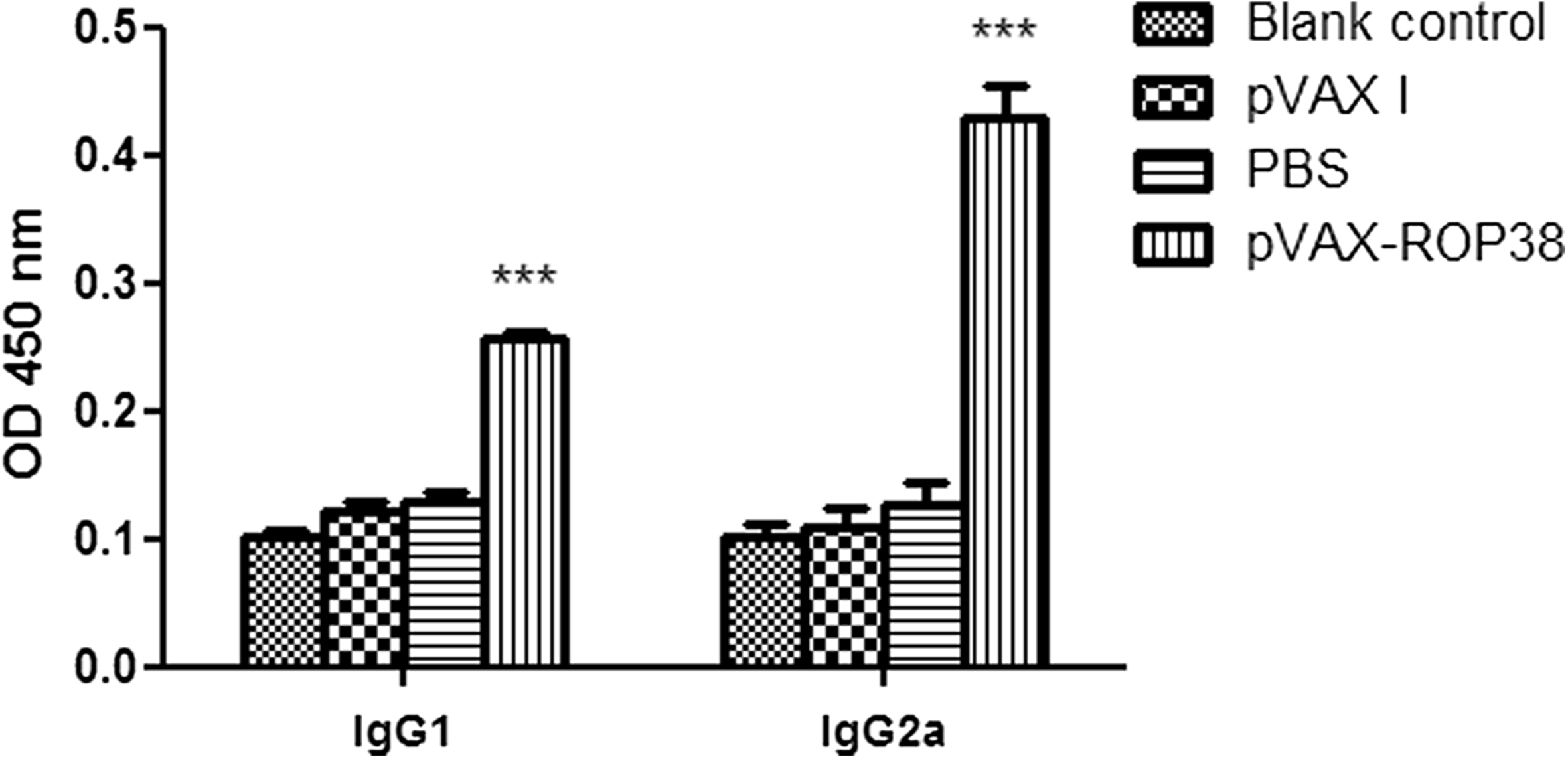

Supplement: Supplementary file 4 — Authors’ original file for figure 4 [file 12879_2014_3845_MOESM4_ESM.tif]

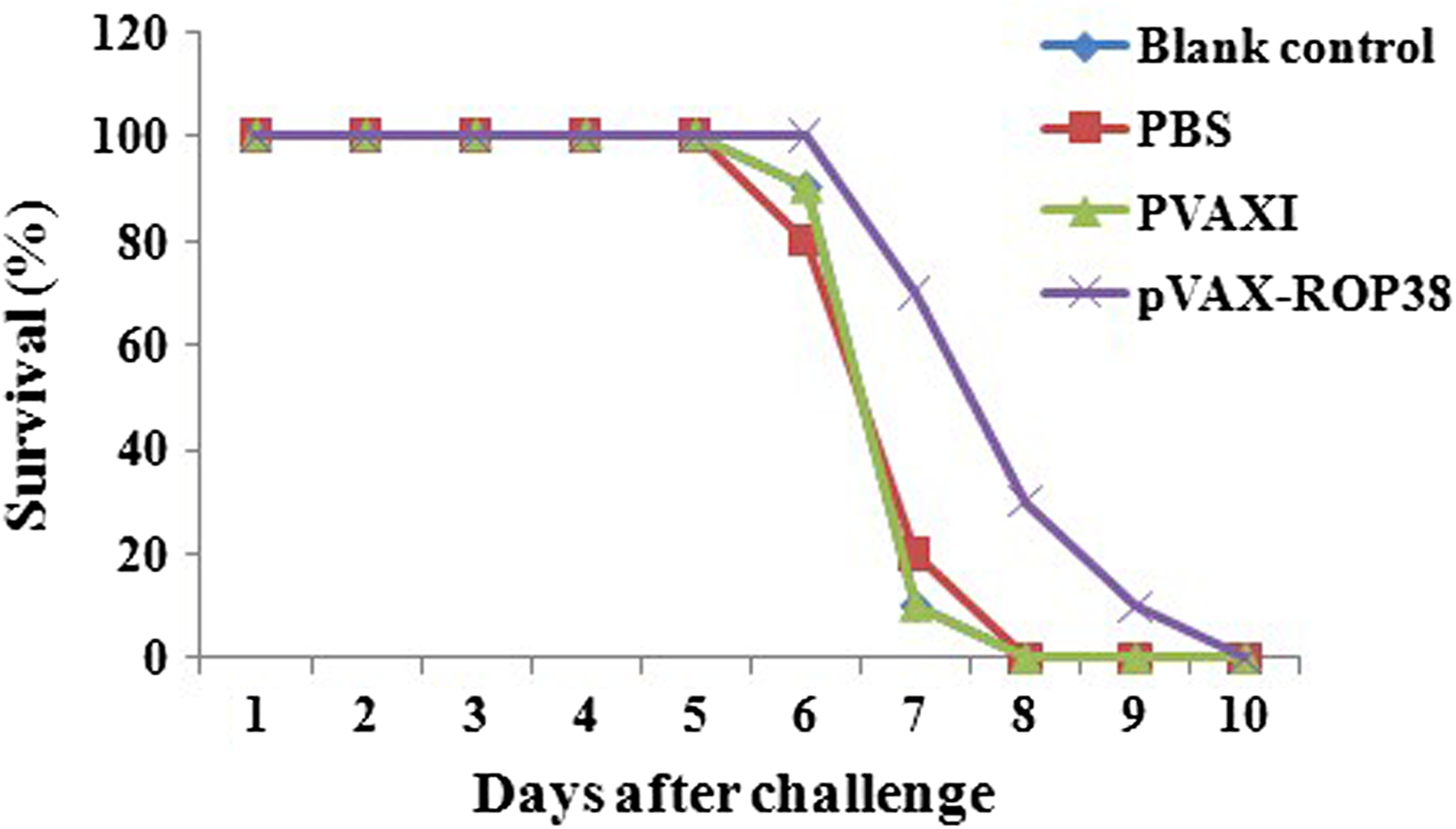

Supplement: Supplementary file 5 — Authors’ original file for figure 5 [file 12879_2014_3845_MOESM5_ESM.tif]
